# Prevalence of gastrointestinal parasites in captive mammals at Khon Kaen Zoo, Thailand

**DOI:** 10.14202/vetworld.2023.2416-2424

**Published:** 2023-12-05

**Authors:** Jirawat Sangpeng, Chatanun Eamudomkarn, Nuttanan Hongsrichan, Atchara Artchayasawat, Chavin Chaisongkram, Kanda Ponsrila, Siriwan Kimkamkaew, Nonglak Laoprom, Thidarut Boonmars, Paiboon Sithithaworn, Opal Pitaksakulrat

**Affiliations:** 1 Department of Parasitology, Faculty of Medicine, Khon Kaen University, Khon Kaen, 40002, Thailand; 2 Department of Research Conservation and Animal Health, Khon Kaen Zoo, 40280, Thailand; 3 Department of General Science, Faculty of Science and Engineering, Kasetsart University, Chalermphrakiat Sakon Nakhon Province Campus, Sakon Nakhon, 47000, Thailand.

**Keywords:** captive mammals, gastrointestinal parasites, zoo, zoonotic parasites

## Abstract

**Background and Aim:**

Captive animals are susceptible to parasitic diseases due to the stress and confinement they experience. In addition, they can serve as reservoirs of zoonotic parasites that have the potential to infect humans. To investigate this possibility, we estimated the prevalence of gastrointestinal (GI) parasites in captive mammals at Khon Kaen Zoo, Thailand.

**Materials and Methods:**

One hundred and forty-seven individual mammals (37 primates, 43 carnivores, 62 herbivores, and 5 rodents) were examined for parasitic infections by fecal examination daily for 3 consecutive days using the formalin-ethyl acetate concentration technique (FECT) and the agar plate culture method.

**Results:**

According to FECT, the overall prevalence of GI parasites was 62.6% (92/147). Within animal groups, the numbers were as follows: 67.6% (25/37) in primates, 23.3% (10/43) in carnivores, 85.5% (53/62) in herbivores, and 80.0% (4/5) in rodents. Using the agar plate culture method, 21.43% (27/126) were positive for *Strongyloides* spp. and hookworm infections. The GI parasites identified belonged to three categories: protozoa (including *Entamoeba histolytica* species complex, *Entamoeba coli*, *Giardia* spp., coccidia, and ciliated protozoa), trematodes (minute intestinal flukes and rumen flukes), and nematodes (strongyle/hookworm, *Strongyloides* spp., Ascarididae, and *Trichuris* spp.).

**Conclusion:**

The findings of this study indicate the prevalence of several GI parasites in zoo animals with the potential for transmission to humans, given the animals’ close proximity to both visitors and animal caretakers.

## Introduction

Zoonotic parasitic diseases can lead to substantial health complications for captive animals [1] and those responsible for their care, such as animal keepers. Exposure to contaminated feces, soil, and plants can put individuals at risk of infection [2, 3]. Helminth infections in captive wild animals can be fatal [4]. Moreover, prolonged captivity can amplify the interaction among parasite species, animals, and humans, increasing the chances of transmission. Protozoa with zoonotic potential that have been detected in captive animals include *Giardia duodenalis*, *Balantioides coli*, *Cryptosporidium* spp., and *Entamoeba histolytica*/*Entamoeba dispar* complex [5, 6]. *Toxoplasma gondii* was recently detected in humans working in a zoo [7]. Many studies have shown that nematode parasites can spread from animals to humans in shared habitats [8]. In particular, non-human primates (NHPs) have a close phylogenetic relationship with humans and can share nematodes such as *Necator americanus, Ancylostoma duodenale, Ascaris lumbricoides, Strongyloides stercoralis, S. fuelleborni, Trichuris trichiura*, and *Enterobius vermicularis* with nearby humans [9–12].

Animal reservoirs frequently release zoonotic parasites into the environment as oocysts, eggs, and larvae in feces [13–16]. Humans can become infected with GI parasites by consuming contaminated food and water containing oocysts or eggs [17–21]. Moreover, direct transmission can occur through contact with the feces of reservoir animals that retain infective larval stages [22]. “One Health” is a worldwide philosophy primarily concerned with the overlooked zoonotic transmission of parasites between animals and humans [23, 24].

Regrettably, despite the risk of zoonotic transmission, little research has addressed the GI parasites in captive wildlife mammals residing within Thailand’s zoos [25, 26]. In response to this knowledge gap, we estimated the prevalence of GI parasites across a range of captive mammals at Khon Kaen Zoo.

## Materials and Methods

### Ethical approval

All animal experiments were approved by the Animal Ethics Committee of the Zoological Park Organization of Thailand (No.2301638) and the Animal Ethics Committee of the Faculty of Medicine, Khon Kaen University (AEMDKKU 004/2022).

### Study period and location

The study was conducted from March to June 2022 in Khon Kaen Zoo, located in Suan Kwang Mountain in Northeastern Thailand (16° 50’ 42.4” N, 102° 53’ 48.1” E). Khon Kaen Zoo was established as an ecotourism and research center for the conservation of rare and threatened species. Enclosures typically contain a covered part, with completely or partially finished floors, and an area exposed to the environment such as a grassy meadow. The animals are fed daily and the enclosures and grounds are cleaned daily, generally in the morning.

### Sample collection and fecal examination

Fecal samples were collected from 147 individual captive mammals housed in Khon Kaen Zoo (37 primates, 43 carnivores, 62 herbivores, and 5 rodents: Table-1). To maximize sensitivity, fecal samples from 3 consecutive days were examined. For 69 individual animals (10 primates, 29 carnivores, and 30 herbivores: Table-2), fecal samples were collected on each of 3 consecutive days. Fresh feces were collected directly from the floor of the enclosures. The sample was retrieved from the center of the fecal mass, packed in plastic bags with the name of the host species, and weighed before being transported in an insulated box (at approximately 15°C) to the laboratory of the Parasitology Department, Faculty of Medicine, Khon Kaen University.

**Table-1:** The numbers of captive mammals from which feces were collected and used for parasite identification in Khon Kaen Zoo, Thailand.

Common name	Species	Number of individuals
Primate		
Chimpanzee	*Pan troglodytes*	4
Bornean Orangutan	*Pongo pygmaeus*	3
Red-shanked Douc Langur	*Pygathrix nemaeus*	3
Hamadryas baboon	*Papio hamadryas*	3
Ring-tailed Lemur	*Lemur catta*	8
Tenasserim Lutung	*Trachypithecus barbei*	1
Common Squirrel Monkey	*Saimiri sciureus*	3
Bengal Slow Loris	*Nycticebus bengalensis*	1
Geoffoy’s Marmoset	*Callithrix geoffroyi*	4
Common Marmoset	*Callithrix jacchus*	6
Golden-handed Tamarin	*Saguinus midas*	1
Total		37
Carnivore		
White Lion	*Panthera leo*	2
Lion	*Panthera leo*	4
Malayan Sun Bear	*Helarctos malayanus*	2
Asiatic Black Bear	*Ursus thibetanus*	4
Binturong	*Arctictis binturong*	4
White Tiger	*Panthera tigris*	1
Indo-Chinese Tiger	*Panthera tigris corbetti*	2
Leopard Cat	*Prionailurus bengalensis*	5
Spotted Hyaena	*Crocuta crocuta*	3
Tanuki	*Nyctereutes procyonoides viverrinus*	4
Small-clawed Otter	*Aonyx cinereus*	2
Asiatic Jackal	*Canis aureus*	4
Common Palm Civet	*Paradoxurus hermaphroditus*	1
Ferret	*Mustela putorius furo*	2
Meerkat	*Suricata suricatta*	1
Fennec fox	*Vulpes zerda*	1
South American Fur Seal	*Arctocephalus australis*	1
Total		43
Herbivore		
Red Kangaroo	*Macropus rufus*	2
white Bennett’s Wallaby	*Macropus rufogriseus*	1
Pygmy Hippopotamus	*Choeropsis liberiensis*	5
Southern White Rhinoceros	*Ceratotherium simum simum*	1
Oryx	*Oryx gazella*	2
Springbok	*Antidorcas marsupialis*	1
Barasingha	*Rucervus duvaucelii*	3
Barbary Sheep	*Ammotragus lervia*	3
Ankole-Watusi	*Bos taurus indicus*	7
Common Barking Deer	*Muntiacus muntjak*	5
Hog Deer	*Axis porcinus*	16
Nyala	*Tragelaphus angasii*	1
Dromedary Camel	*Camelus dromedarius*	1
Nilgai	*Boselaphus tragocamelus*	1
Chinese Serow	*Capricornis milneedwardsii*	1
Burchell’s Zebra	*Equus quagga burchellii*	2
Giraffe	*Giraffa camelopardalis*	2
Spotted Deer	*Axis axis*	1
Rusa Deer	*Rusa timorensis*	2
Elephant	*Elephas maximus*	1
Brow-Antlered Deer	*Rucervus eldii thamin*	3
Sika Deer	*Cervus nippon*	1
Total		62
Rodentia		
Malayan Porcupine	*Hystrix brachyura*	2
Capybara	*Hydrochoerus hydrochaeris*	3
Total		5
All samples		147

**Table-2:** The numbers of captive mammals from which feces were collected on each of 3 consecutive days and used for parasite identification in Khon Kaen Zoo, Thailand.

Common name	Species	Number of individual
Primate		
Bornean Orangutan	*Pongo pygmaeus*	3
Red-shanked Douc Langur	*Pygathrix nemaeus*	1
Ring-tailed Lemur	*Lemur catta*	3
Tenasserim Lutung	*Trachypithecus barbei*	1
Common Marmoset	*Callithrix jacchus*	1
Golden-handed Tamarin	*Saguinus midas*	1
Total		10
Carnivore		
White Lion	*Panthera leo*	1
Lion	*Panthera leo*	4
Malayan Sun Bear	*Helarctos malayanus*	2
Asiatic Black Bear	*Ursus thibetanus*	4
Binturong	*Arctictis binturong*	1
White Tiger	*Panthera tigris*	1
Indo-Chinese Tiger	*Panthera tigris corbetti*	2
Leopard Cat	*Prionailurus bengalensis*	1
Spotted Hyaena	*Crocuta crocuta*	3
Tanuki	*Nyctereutes procyonoides viverrinus*	4
Asiatic Jackal	*Canis aureus*	4
Common Palm Civet	*Paradoxurus hermaphroditus*	1
South American Fur Seal	*Arctocephalus australis*	1
Total		29
Herbivore		
Red Kangaroo	*Macropus rufus*	2
White Bennett’s Wallaby	*Macropus rufogriseus*	1
Pygmy Hippopotamus	*Choeropsis liberiensis*	1
Southern White Rhinoceros	*Ceratotherium simum simum*	1
Oryx	*Oryx gazella*	2
Springbok	*Antidorcas marsupialis*	1
Ankole-Watusi	*Bos taurus indicus*	7
Common Barking Deer	*Muntiacus muntjak*	5
Hog Deer	*Axis porcinus*	2
Nyala	*Tragelaphus angasii*	1
Dromedary Camel	*Camelus dromedarius*	1
Nilgai	*Boselaphus tragocamelus*	1
Chinese Serow	*Capricornis milneedwardsii*	1
Burchell’s Zebra	*Equus quagga burchellii*	1
Giraffe	*Giraffa camelopardalis*	2
Elephant	*Elephas maximus*	1
Total		30
All samples		69

### Formalin-ethyl acetate concentration technique (FECT)

All fecal samples were analyzed using the FECT [27] with 3 consecutive days’ examinations of each sample (a total of 354 samples). Two grams of feces were mixed with 10 mL of 10% formalin solution, filtered into a 15 mL centrifuge tube using two layers of gauze, and centrifuged at 500× g for 5 min. After removing the supernatant, the debris was mixed with 3 mL of ethyl acetate solution and 7 mL of 10% formalin solution and centrifuged at 500 *g* for 5 min. After removing the supernatant, 1 mL of 10% formalin solution was added to the sediment. Two drops of the three aliquots were stained with 1% iodine and examined under a light microscope at 10× and 40× magnifications (Olympus, Japan). Parasites were identified based on eggs’ color, shape, and content or the anatomy of trophozoites, larvae, or other propagules [28, 29].

### Agar plate culture technique (APCT)

*Strongyloides* spp. and hookworms were detected using an APCT. A total of 126 fecal samples (each approximately 2 g) were available for examination by APCT. Filariform larvae of *Strongyloides*, hookworms, and free-living adults of *Strongyloides* were investigated after 4-5 days of culture at room temperature (27°C–35°C).

### Statistical analysis

The percentage of individuals infected with each species of parasite was calculated. McNemar’s Chi-square test was used to compare proportions from paired samples [30] and to determine whether the ability to detect a parasite from a single fecal sample was significantly different from that based on samples from 3 consecutive days. Statistical analysis was considered significant at p < 0.05.

## Results

The overall prevalence of GI parasites was 62.6% (92/147) in captive mammals at Khon Kaen Zoo, Thailand, according to the FECT. Corresponding values for different groups of mammals were as follows: primates, 67.6% (25/37); carnivores, 23.3% (10/43); herbivores, 85.5% (53/62); and rodents, 80.0% (4/5) (Table-3). In addition, 126 fecal samples were examined using the APCT and 21.43% (27/126) were positive for *Strongyloides* or hookworm (Table-4).

**Table-3:** The overall prevalence of GI parasites in captive mammals according to the FECT.

Type	Number of animals	Parasite positive (%)
Primate	37	25 (67.6)
Carnivore	43	10 (23.3)
Herbivore	62	53 (85.5)
Rodents	5	4 (80.0)
Total	147	92 (62.6)

GI=Gastrointestinal, FECT=Formalin-ethyl acetate concentration technique

**Table-4:** The prevalence of GI parasites in captive mammals according to the APCT.

Types	Number	*Strongyloides* spp. (%)	Hookworm (%)	Mixed infection (%)
Primate	21	4 (19.0)	4 (19.0)	4 (19.0)
Carnivore	38	-	3 (7.9)	-
Herbivore	62	3 (4.9)	7 (11.3)	-
Rodent	5	2 (40.0)	-	-
Total	126	9 (7.1)	14 (11.1)	4 (3.2)

GI=Gastrointestinal, APCT=Agar plate culture technique

**Figure-1:**
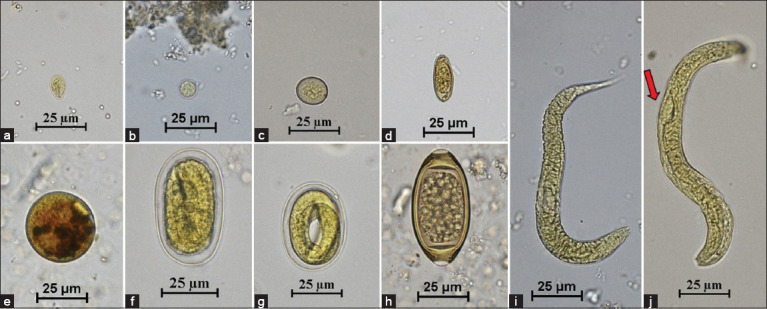
Figures of gastrointestinal parasites in fecal samples of captive primates. (a) *Giardia* spp. (40×); (b) *Entamoeba histolytica* species complex (40×); (c) *Entamoeba coli* (40×); (d) Minute intestinal trematode (40×); (e) Ciliated protozoa (40×); (f) Hookworm (40×); (g) *Strongyloides* spp. (40×); (h) *Trichuris* spp. (40×); (i) Hookworm rhabditiform larva (40×); and (j) *Strongyloides* spp. rhabditiform larva (40×). Red arrow = Prominent genital primordium.

**Figure-2:**
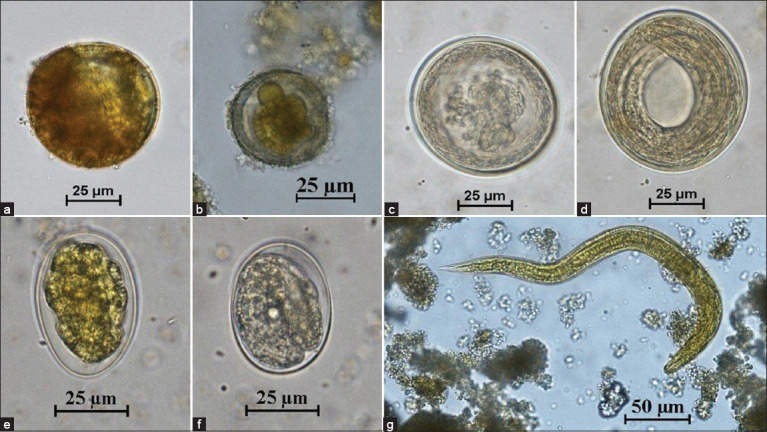
Figures of gastrointestinal parasites in fecal samples of captive carnivores. (a) Ciliated protozoa (40×); (b-d) Ascarididae (40×); (e) Hookworm (40×); (f) *Strongyloides* spp. (40×); and (g) Hookworm rhabditiform larva (40×).

**Figure-3:**
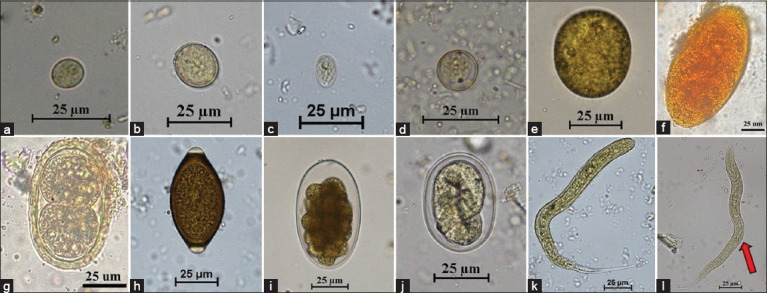
Figures of gastrointestinal parasites in fecal samples of captive herbivores. (a) *Entamoeba histolytica* species complex (60×); (b) *Entamoeba coli* (40×); (c) *Giardia* spp. (40×); (d) Coccidia cyst (60×); (e) Ciliated protozoa (40×); (f) Rumen fluke (40×); (g) Ascarididae (40×); (h) *Trichuris* spp. (40×); (i) Strongyle (40×); (j) *Strongyloides* spp. (40×); (k) Strongyle rhabditiform larva (40×); and (l) *Strongyloides* spp. rhabditiform larva (40×). Red arrow = prominent genital primordium.

The prevalence of GI parasites in 69 individual animals was determined by examination of fecal samples collected on 3 consecutive days. The prevalence rates were 55.1% (38/69), 49.28% (34/69), and 52.17% (36/69) based on the 1^st^-, 2^nd^-, and 3^rd^-day examination, respectively. One new infected individual was detected on the 2^nd^ day of examination, and three on the 3^rd^ day (Table-5). The McNemar test showed no statistically significant differences between day 2 (p > 0.05) and the cumulative 3 consecutive days (p > 0.05). The GI parasites found in captive primates included *Giardia* spp., *E. histolytica* species complex, *Entamoeba coli*, minute intestinal trematodes, ciliated protozoa, hookworm, *Strongyloides* spp., and *Trichuris* spp. In carnivores, the GI parasites included ciliated protozoa, Ascarididae, hookworm, and *Strongyloides* spp. In herbivores, the feces yielded *E. histolytica* species complex, *E. coli*, *Giardia* spp., coccidia cysts, ciliated protozoa, rumen flukes, Ascarididae, *Trichuris* spp., strongyles, and *Strongyloides* spp. (Figures-1–3). Rodents had *E. histolytica* species complex, hookworms, *Strongyloides* spp., and *Trichuris* spp.

**Table-5:** The frequency of detection of GI parasites in captive mammals on 3 consecutive days according to the FECT.

Type/day	Number	Parasite positive on each day (%)	New individual discovery
Mammal			
Day 1	69	38 (55.07)	-
Day 2	69	34 (49.28)	1
Day 3	69	36 (52.17)	3
Total	69	42 (60.87)	4

GI=Gastrointestinal, FECT=Formalin-ethyl acetate concentration technique

## Discussion

In our study, fecal samples were collected directly from the floor of zoo animal enclosures. This non-invasive approach eliminates the need for chemical or mechanical restraint of the animals, thus reducing stress and not affecting their welfare [31, 32]. The significant finding of a considerable range of GI parasitic infections (62.6% of animals infected) among captive mammals at Khon Kaen Zoo raises concerns. Comparable prevalence values have been reported from zoo populations in other countries, such as Nepal (19.5%) [33], Malaysia (56.3%) [34], and Bangladesh (60.5%) [35]. Higher prevalence has been reported in some cases, such as 68.3% in the Rio de Janeiro Zoo [36], 72.5% in Spain [5], and 71.8% and 74.2% in Brazil [37, 38].

Identifying protozoans within the captive animal in our study reveals the potential for easy transmission among hosts due to the environmental resilience of cysts and oocysts, capacity for passive oral transmission, and lack of requirement for intermediate hosts [5, 6]. Most parasite infections in wild animals are asymptomatic [39], but stress from captivity can make them symptomatic, resulting in severe clinical symptoms of diarrhea [40–42]. We detected *Giardia* spp., *E. histolytica* species complex, hookworms, *Strongyloides* spp., Ascarididae, and *Trichuris* spp., all of which have the potential for transmission in the zoo environment. GI parasites can spread to animal keepers, who may not always be aware of the risk [43–45]. Prevention of transmission requires a multifaceted approach encompassing suitable medications, food-handling practices, and heightened sanitation to enhance animal and worker welfare. Contaminated food and water are the major sources of GI parasite infections and are likely the transmission routes of infections that we detected in this study.

The quantity of stool samples adequate to detect intestinal parasites in epidemiologic research is still uncertain [46]. Parasites can produce eggs or cysts intermittently, which means that a single fecal examination may not detect all cases. Although traditional fecal examination techniques, including the APCT, FECT, Baermann technique, and direct smear, have been the main reference procedures for diagnosing strongyloidiasis, these techniques have low sensitivity and are unreliable due to irregular larval excretion in humans [47, 48] and the high fluctuation in larval excretion from animals [49]. Hence, examination of fecal samples collected on multiple days could improve the accuracy of detecting parasites, making it possible to provide adequate treatment in a timely manner. It is typically recommended to examine stool samples collected on 3 different days [50], an approach demonstrated to improve the detection of organisms such as *E. histolytica*/*E. dispar* [51, 52]. Collection of fecal samples in the zoo is quite easy due to the high compliance of organization, routine cleaning, and regular use of anthelmintic treatments.

A previous study by Moustafa [53] showed that using three consecutive daily examinations, sensitivity of the agar plate method increased from 70.3% to 96.2%. Another study revealed a significantly higher cumulative positive rate of *S. stercoralis* from 13.3% to 22% by examining fecal samples daily for 3 consecutive days [54]. Repeated fecal examinations clearly increase the evaluation of the prevalence of strongyloidiasis, which is an important disease in humans.

However, the findings of this study imply that the prevalence of GI parasites acquired through a single stool examination using the FECT technique could be equally reliable when compared to the results from the analysis of fecal samples collected over 3 consecutive days. Importantly, it should be noted that the parasites identified in this investigation potentially have the capacity for zoonotic transmission due to their hosts’ close proximity to humans.

One limitation of this study was the problem of fecal collection from known individuals of herd animals such as many herbivores. It can be difficult to identify the feces of each animal in a group, particularly if they are free-ranging or have access to shared feeding and watering areas. In these circumstances, the collection process can be time-consuming and labor-intensive, especially when dealing with a large group. Furthermore, this procedure may induce stress among the animals, leading to potential alterations in their behavior and defecation patterns. Finally, variations might increase as some animals defecate more frequently or in different locations than others, consequently challenging the accuracy of individual fecal collection to reflect the overall herd prevalence. The results from this study will provide information on the prevalence of parasitic infection in captive mammals and hence inform zoo management to improve animal welfare and health. It is important to minimize the dangers of zoonotic infections to tourists, researchers, animal keepers, and veterinarians.

## Conclusion

To the best of our knowledge, this study is the first to investigate the prevalence of GI parasites in captive mammals kept in the Khon Kaen Zoo (includes NHPs, carnivores, herbivores, and rodents) based on examination of fecal samples on each of 3 consecutive days. The zoo animals served as important reservoir hosts for several zoonotic GI parasites such as *Giardia* spp., *E. histolytica* species complex, hookworms, *Strongyloides* spp., Ascarididae, and *Trichuris* spp. These parasites possess the capacity to propagate among animal hosts, potentially triggering disease, and representing a hazard to zookeepers, veterinarians, and visitors at Khon Kaen Zoo. The key is to implement the prevention and control of these GI parasites. This calls for a One Health approach to ensure the well-being of animals, caretakers, and visitors.

## Authors’ Contributions

JS: Methodology, investigation, data analysis, and writing-original draft. CE, NH, and AA: Methodology, validation, and writing-review and editing. CC, KP, and SK: Investigation and writing-review and editing. NL: Validation and writing-review and editing. TB and PS: Conceptualization, methodology, and writing-review and editing. OP: Conceptualization, methodology, validation, investigation, data analysis, and writing-original draft. All authors have read, reviewed, and approved the final manuscript.
